# Luteolin Alleviates Ulcerative Colitis in Mice by Modulating Gut Microbiota and Plasma Metabolism

**DOI:** 10.3390/nu17020203

**Published:** 2025-01-07

**Authors:** Shuai Yang, Hongwei Duan, Zhenxing Yan, Chen Xue, Tian Niu, Wenjing Cheng, Yong Zhang, Xingxu Zhao, Junjie Hu, Lihong Zhang

**Affiliations:** 1 College of Veterinary Medicine, Gansu Agricultural University, Lanzhou 730070, China; yangshuai11200@163.com (S.Y.); grand6138@163.com (H.D.);; 2 Gansu Key Laboratory of Animal Generational Physiology and Reproductive Regulation, Lanzhou 730070, China

**Keywords:** gut microbiota, inflammatory bowel disease, Luteolin, plasma metabolism, ulcerative colitis

## Abstract

Background/Objectives: Ulcerative colitis (UC) is a chronic and easily recurrent inflammatory bowel disease. The gut microbiota and plasma metabolites play pivotal roles in the development and progression of UC. Therefore, therapeutic strategies targeting the intestinal flora or plasma metabolites offer promising avenues for the treatment of UC. Luteolin (Lut), originating from a variety of vegetables and fruits, has attracted attention for its potent anti-inflammatory properties and potential to modulate intestinal flora. Methods: The therapeutic efficacy of Lut was evaluated in an established dextran sodium sulfate (DSS)-induced colitis mice model. The clinical symptoms were analyzed, and biological samples were collected for microscopic examination and the evaluation of the epithelial barrier function, microbiome, and metabolomics. Results: The findings revealed that Lut administration at a dose of 25 mg/kg significantly ameliorated systemic UC symptoms in mice, effectively reduced the systemic inflammatory response, and significantly repaired colonic barrier function. Furthermore, Lut supplementation mitigated gut microbiota dysbiosis in a UC murine model, increasing the abundance of *Muribaculaceae*, *Rikenella*, and *Prevotellaceae* while decreasing *Escherichia_Shigella* and *Bacteroides* levels. These alterations in gut microbiota also influenced plasma metabolism, significantly increasing phosphatidylcholine (PC), 6′-Deamino- 6′-hydroxyneomycin C, and gamma-L-glutamyl-butyrosine B levels and decreasing Motapizone and Arachidoyl-Ethanolamide (AEA) levels. Conclusions: This study reveals that Lut supplementation modulates intestinal inflammation by restoring the gut microbiota community structure, thereby altering the synthesis of inflammation-related metabolites. Lut is a potential nutritional supplement with anti-inflammatory properties and offers a novel alternative for UC intervention and mitigation. In addition, further studies are needed to ascertain whether specific microbial communities or metabolites can mediate the recovery from UC.

## 1. Introduction

Inflammatory bowel disease (IBD), a chronic inflammatory disease of the intestine, is caused by immune system abnormalities and can present as ulcerative colitis (UC) and Crohn’s disease (CD) [1,2]. The main clinical manifestations of UC include chronic diarrhea, abdominal pain, and rectal bleeding, all of which pose significant threats to human health [3]. Currently, the treatment options for UC primarily involve medications, such as steroids, immunosuppressants, and antibiotics [4]. However, these medications do not completely cure UC and can lead to serious side effects [5]. With advances being made in medical technology, there is an increasing demand for safer and more effective treatments. Indeed, newer drugs, such as biologics, have shown promise in clinical practice [6]. Consequently, there is an urgent need to develop new, safe, cost-effective, and efficient methods for treating UC.

The precise pathogenesis of UC remains unclear; however, it is thought to be closely related to factors such as immune response dysregulation, defective epithelial barriers, and genetic susceptibility [7]. Environmental triggers, particularly dietary imbalances, may disrupt the ecological balance of the gut flora, contributing significantly to UC pathogenesis [8]. The gut microbiota play an important role in various host functions, including food digestion and absorption, barrier integrity maintenance, and immune and metabolic regulation [9,10]. Studies have demonstrated that microbiota can migrate from the lumen to the intestinal lamina propria, stimulating the release of interleukin (IL)-1, IL-6, and IL-23, which drives pathogenic type 17 helper T cell responses and ultimately immune cell recruitment [11]. Excessive immune responses can lead to abnormal tight junctions (TJs) in intestinal epithelial cells and compromised barrier function, primarily due to the altered subcellular distribution and expression of TJ proteins [12]. Once the intestinal barrier is damaged, microbiota imbalances can trigger a series of immune responses, further disrupting TJ and barrier functions, thereby exacerbating intestinal inflammation and perpetuating a vicious cycle [13]. Consequently, the regulation of intestinal flora disorders and the amelioration of impaired intestinal barrier function have become rational strategies for the prevention and treatment of UC.

Disturbances have been observed in the gut microbiota among patients with UC and in animal models of enterocolitis [14]. Specifically, patients with UC have been found to exhibit reduced diversity in the gut microbiota, with a decrease in the relative abundance of *Firmicutes* and an increase in the relative abundances of *Proteobacteria* and *Enterobacteriaceae* [15,16]. Interventions involving the modulation of the gut microbiota through the administration of drugs and beneficial bacteria have been proven effective in mitigating intestinal inflammation [17]. Furthermore, certain biomolecules produced by microorganisms, such as short-chain fatty acids (SCFAs), exert anti-inflammatory effects. These SCFAs can influence the number and activity of intestinal T regulatory cells and inhibit the release of pro-inflammatory cytokines by macrophages [18]. Additionally, tryptophan, bile acids, and their metabolites contribute to the improvement of intestinal microbiota disorders and enhance intestinal barrier function [19]. Small molecular compounds in plasma serve as terminal points of the biometabolic chain and exhibit signal amplification effects [20]. Therefore, studying the changes in plasma metabolites during the pathogenesis and treatment of UC may provide valuable insights into the mechanisms underlying the disease and identify novel therapeutic targets for drug development.

In recent years, the potential benefits of traditional Chinese medicine and its monomer components, which are known for their low toxicity, have become of interest in studies on the prevention and treatment of UC. Notably, the Pulsatilla decoction has demonstrated efficacy in alleviating UC by restoring intestinal barrier integrity and regulating intestinal SCFA metabolism [21]. Additionally, Rhein, the main component of rhubarb, has shown promise in improving experimental colitis by regulating the intestinal flora and host purine metabolism [22]. Luteolin (Lut), an active ingredient in medicinal materials such as *Lonicerae japonicae* flos and chrysanthemum, as well as in vegetables such as Brussels sprouts and green peppers, exhibits pharmacological properties including anti-inflammatory, anti-tumor, anti-oxidation, and immune regulatory effects [23,24,25]. Specifically, Lut has been shown to inhibit inflammatory responses by reducing the expression of the pro-inflammatory cytokines tumor necrosis factor (TNF)-α, IL-1β, and IL-6 and has demonstrated efficacy in the treatment of neutrophilic asthma, lupus nephritis, and Alzheimer’s disease [26,27,28]. Despite previous studies indicating that Lut plays a role in the prevention and treatment of UC, the precise mechanisms through which it affects UC by regulating the intestinal microbiota and body metabolism remain unclear. Therefore, the aim of this study is to explore the potential therapeutic effects of Lut in a DSS-induced UC mouse model and further elucidate its specific mechanisms of action, based on a comprehensive analysis of the gut microbiome and metabolome.

## 2. Materials and Methods

### 2.1. Animal Model

Male BALB/c mice, weighing 18–22 g and aged 8 weeks, were obtained from the Experimental Animal Center at the Lanzhou Veterinary Research Institute, Chinese Academy of Agricultural Sciences (permit number: SCXK (Gan) 2020–0002). The animals were housed in a standard environment (with a 12 h light/dark cycle, temperature of 23 ± 1 °C, and relative humidity of 45 ± 5%) for a one-week acclimatization period, during which they had unrestricted access to both distilled water and a commercial diet for rodents.

Mice were randomly assigned to three groups (totaling 24; 8 per group) using a random number table: control, DSS (DSS with a molecular weight of 36,000–50,000 Da; MP Biomedicals, Santa Ana, CA, USA), and Lut (MedChem Express, Princeton, NJ, USA) [29]. The Lut groups were administered 25 mg/kg of Lut dissolved in 5% sodium hydroxymethylcellulose (CMC-Na). The choice of Lut dosage was based on our preliminary experiments, which were also justified by relevant studies [30,31]. The control and DSS groups were administered equal volumes of CMC-Na via gavage at a volume of 0.1 mL per 10 g of body weight once daily for 14 days. From days 8 to 14, the DSS and Lut groups were provided a daily supply of 3% (*w*/*v*) DSS dissolved in distilled water; the control group was excluded from this treatment [32].

### 2.2. Data and Sample Collection

Daily assessments were conducted throughout the experiment to record the presence of fecal occult blood, body weight, and fecal viscosity, which were used to calculate the disease activity index (DAI) [33]. The total DAI score ranges from 0 to 12, with higher scores indicating more severe symptoms. Table 1 provides the detailed assessment criteria for the DAI. On day 14, the mice were anesthetized and euthanized. Blood samples were collected for serum separation and subjected to enzyme-linked immunosorbent assay (ELISA) analyses, and plasma was collected for metabolomic profiling. The length of the intestinal tract was measured from the cecum to the anal opening, and colonic tissue samples were collected for histopathological analyses and protein and RNA extraction. The spleen was weighed to calculate the spleen index (spleen weight/body weight), and the blind and colonic contents were collected for 16S rRNA sequencing to assess microbial diversity.

### 2.3. Histopathological Analysis

Tissue samples from the colon were preserved using 4% paraformaldehyde and then embedded in paraffin wax and subsequently sliced into sections. Goblet cell functionality was assessed through periodic acid–Schiff (PAS) staining, whereas tissue injury was examined by hematoxylin–eosin (H&E) staining. Independent histological scoring of inflammation was performed by two senior pathologists in a double-blind manner. The detailed evaluation criteria are listed in Table S1. In brief, the severity of inflammation and colonic tissue damage were ranked on a scale of 0 to 4, and their combined total score constituted the histological score [34].

### 2.4. Real-Time qPCR Validation

RNA extraction and qPCR were performed according to the methodologies outlined in our previous study [35]. The primer sequences used for validation are listed in Table S2.

### 2.5. Immunohistochemistry

Deparaffinized sections of 4 μm thickness were placed on gelatin/polylysine-coated glass slides and dried in an incubator at 60 °C for 2 h. They were then deparaffinized twice in xylene for 15 min each. Subsequently, the sections were soaked in ethanol solutions of different concentrations (100%, 90%, and 70% (*v*/*v*)) and finally dewaxed in water. They were then washed three times with 0.01 M phosphate-buffered saline (PBS) (pH 7.4) for 3 min per wash. To block endogenous peroxidase activity, the sections were incubated in 0.3% H2O2 (*w*/*v*) for 10 min. The anti-zonulaoccludens-1 (ZO-1) antibody (21773-1-AP, Proteintech, Wuhan, China) and anti-Occludin antibody (66378-1-Ig, Proteintech) were diluted to 1:300 and incubated with the sections. The coloration was developed with diaminobenzidine, and the nuclei were stained with hematoxylin as a counterstain. The prepared sections were then observed under an Olympus-DP73 light microscope (Olympus, Tokyo, Japan) [36].

### 2.6. Enzyme-Linked Immunosorbent Assay

Mouse sera were collected without dilution for the assay. The levels of TNF-α (YM-S2863, Yuanmu, Shanghai, China), IL-6 (YM-S2894, Yuanmu), IL-1β (YM-S2771, Yuanmu), and IL-10 (YM-S2907, Yuanmu) cytokines were measured using ELISA kits, following the recommendations of the manufacturer. The absorbance values for each sample were measured repeatedly at 450 nm, and the absorbance values of the negative control wells containing the reaction solution, but not the sample, were subtracted. The coefficient between the intraplate and interpolated variations was less than 15%. The lowest detectable concentration for all cytokines was less than 1.0 pg/mL.

### 2.7. Western Blot

Western blot was performed as previously described. Colon tissues were washed in pre-cooled PBS and lysed using radioimmunoprecipitation assay (RIPA) buffer (Solarbio, Beijing, China) supplemented with 1 mM phenylmethylsulfonyl fluoride (PMSF) (Solarbio). Proteins from each group were subjected to polyacrylamide gel electrophoresis and transferred onto a 0.45 μm PVDF membrane (Millipore, Bedford, MA, USA) in equal quantities. The transferred membranes were subsequently incubated for 1 h at 25 °C in Tris-buffered saline supplemented with 0.1% Tween-20 and 5% BSA to block non-specific binding. Following blocking, the membranes were probed with the respective primary antibodies overnight at 4 °C. Anti-ZO-1 (66452-1-Ig, 1:500, Proteintech, Wuhan, China), anti-Occludin (66378-1-Ig, 1:2000, Proteintech), anti-Claudin-1 (28674-1-AP, 1:1000, Proteintech), anti-IL-6 (26404-1-AP, 1:500, Proteintech), anti-TNF-α (17590-1-AP, 1:500, Proteintech), anti-cyclooxygenase-2 (COX-2) (66351-1-Ig, 1:1000, Proteintech), anti-IL-1β (16806-1-AP, 1:500, Proteintech), and anti-β-actin (bs-0061R, 1:3000, Bioss, Beijing, China). After being washed, the membranes were incubated with horseradish peroxidase-conjugated goat anti-rabbit or anti-mouse IgG (SA00001-2, 1:5000, Proteintech) for 1 h at 37 °C. The blots were visualized using a supersensitive ECL chemiluminescent substrate (Biosharp, Hefei, China) and the Amersham Imager 600 chemiluminometer (GE Healthcare Biosciences, Boston, MA, USA). A grayscale analysis of the Western blot strips was performed using ImageJ software (version 1.4.3), with the grayscale values normalized to the fold change in the control and β-actin as a loading control.

### 2.8. Gut Microbiota Analysis

Nucleic acids were extracted using the TGuideS96MagneticSoil/Stool DNA Kit (Tiangen Biotech Co., Ltd., Beijing, China). The gut bacterial composition was determined via the amplification and sequencing of the V3-V4 region of bacterial 16S rRNA. The specific primers used were F: 5′-AGRGTTTGATYNTGGCTCAG-3′ and R: 5′-TASGGHTACCTTGTTASGACTT-3′. The amplicons were sequenced on an Illumina MiSeq PE300 platform (Illumina, San Diego, CA, USA). The sequencing results were analyzed using Usearch 10.0.240_i86 software, and operational taxonomic units (OTUs) were obtained by clustering at a 97.0% similarity level. Based on the SILVA ribosomal RNA database, the naive Bayes classifier combined with the alignment method was used to annotate the feature sequences, enabling the identification of the corresponding species classification information of each feature. Alpha diversity was calculated and presented using QIIME2 2020.6.0 and R. Beta diversity was determined using QIIME 2020.6.0. A principal component analysis (PCA) diagram was drawn using R tools based on beta diversity. The linear discriminant analysis (LDA) effect size (LEfSe) was performed to find the differentially characterized microorganisms (LDA score ≥ 4, *p*-value < 0.05). Spearman’s correlation analysis was used to observe the correlation between bacteria and clinical indicators in UC mice.

### 2.9. Analysis of Plasma Metabolomics

To prepare the plasma samples for a metabolomic analysis, 500 μL of extract (methanol/acetonitrile = 1:1, internal standard concentration 2 mg/L) containing the internal standard (1000:2) was added to 100 μL of plasma. The mixture was vortexed for 30 s to precipitate the proteins. Following an ice bath at –20 °C for 1 h, the supernatant was collected using centrifugation at 500× *g* for 15 min. The extracts were then dried in a vacuum concentrator and purified again. The supernatant was decanted, and 10 μL of each sample was blended with quality control (QC) samples for subsequent instrumental analysis. For the liquid chromatography–mass spectrometry (LC-MS) analysis, an Acquity I-Class PLUS coupled with a Xevo G2-XS QTof high-resolution mass spectrometer (Waters, Milford, CT, USA) was employed, equipped with an Acquity UPLC HSS T3 column (1.8 μm, 2.1 × 100 mm). The LC-MS parameters for both positive and negative ion modes included the following: mobile phase A, 0.1% formic acid in water; mobile phase B, 0.1% formic acid in acetonitrile; sample injection volume, 1 μL; elution gradient, starting at 2% B for 0–0.25 min, increasing to 98% B for 0.25–13.0 min, and returning to 2% B for 13.0–15.0 min, with a flow rate of 0.4 mL/min. The mass spectrometer settings were as follows: capillary voltage, 2000 V for positive ion mode and –1500 V for negative ion mode; cone voltage, 30 V; ion source temperature, 150 °C; desolvation temperature, 500 °C; back blowing flow rate, 50 L/h; and desolvation gas flow rate, 800 L/h.

### 2.10. Statistical Analysis of Plasma Metabolomics

Metabolomics data analysis was conducted after removing two outliers from each group, with *n* = 6 per group. The initial data acquired with MassLynx V4.2 were processed, involving peak detection and alignment, by utilizing Progenesis QI software (version 2.0). The identification of theoretical fragments was conducted via the METLIN database accessible within Progenesis QI, with a mass accuracy tolerance of 100 ppm. To prepare for further analysis, the raw peak areas were normalized to the sum of all peak areas. Principal component analysis (PCA) and Spearman’s correlation analysis were employed to evaluate the consistency of the results within the group. Subsequently, the detected compounds were queried in the Kyoto Encyclopedia of Genes and Genomes (KEGG) database to obtain information on their classification and associated metabolic pathways. Based on the classification information, the fold change between the compounds was calculated and compared. The significance of each compound was evaluated using a *t*-test to determine the compound *p* value. Orthogonal Partial least squares discriminant analysis (OPLS-DA) was performed using R software (version 4.2.1). The model’s dependability was confirmed through the execution of two hundred permutation tests. Additionally, the variable importance in projection (VIP) values were computed using repeated cross-validation procedures to assess the model’s efficacy. Differential compounds were screened using a combination of the fold change, *p* value, and VIP values obtained from the OPLS-DA model. The screening criteria were set as follows: fold change ≥ 1, *p* value < 0.05, and VIP ≥ 1. Finally, the significance of the differential metabolite enrichment in the KEGG pathway analysis was calculated using a hypergeometric distribution test, which provided insights into the biological pathways affected by the identified differential compounds.

### 2.11. Statistical Analysis

The statistical analysis of the data was conducted with SPSS software (version 21.0, SPSS Inc., Chicago, IL, USA). For comparing multiple groups, a one-way analysis of variance (ANOVA) test was applied. The results are presented as mean ± standard deviation (SD), with statistical significance set at *p* < 0.05. GraphPad Prism software (version 9.0) was used for data visualization. Correlation analyses were performed using ChiPlot (https://www.chiplot.online, accessed on 29 August 2024), which provided a clear and informative graphical representation of the relationships between the variables.

## 3. Results

### 3.1. Luteolin Exerts Protective Effects Against DSS-Induced Colitis in Mice

**Figure 1:**
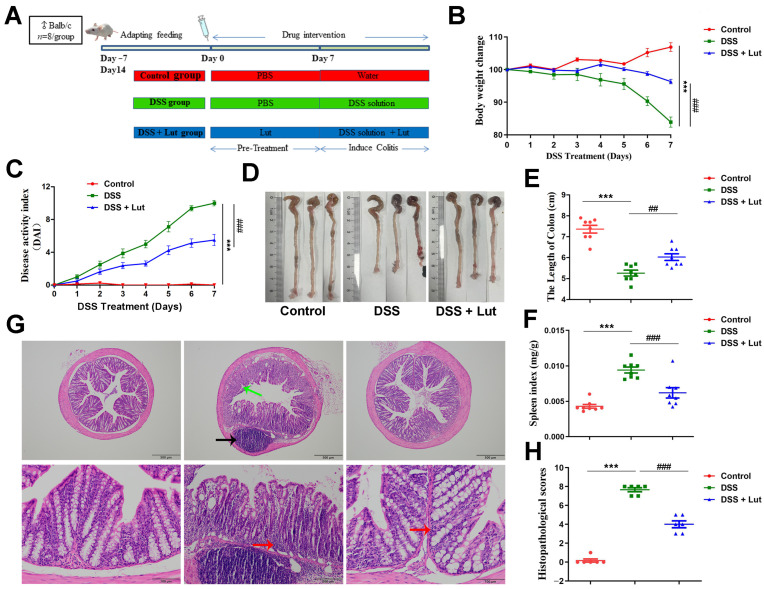
Protective effect of Luteolin on DSS-induced colitis in mice. (**A**) Schematic illustrating the DSS-induced mouse colitis model and Lut intervention. (**B**) Percent changes in the body weights of the mice relative to those on day 1. (**C**) DAI scores based on weight loss, stool consistency, and bleeding evaluation. (**D**) Representative picture of a colon. (**E**) Length of a colon. (**F**) Spleen index in mice. (**G**) Histological examination was performed on sections of the colon that were stained with H&E. Neutrophil infiltration is denoted by the black arrow, transmural inflammation accompanied by the disruption of crypt architecture is marked by the green arrow, and inflammatory cell infiltration is indicated by the red arrow (upper row, 10×, scale bar = 500 μm; lower row, 40×, scale bar = 100 μm). (**H**) Histological scores based on H&E staining (*n* = 6). The data are expressed as mean ± SD (*n* = 8). *** *p* < 0.001 vs. the control group; ## *p* < 0.01, ### *p* < 0.001 vs. the DSS group.

To investigate the potential therapeutic effects of Lut on the development of UC, a DSS-induced mouse colitis model was established (Figure 1A). The DSS-treated mice exhibited systemic symptoms, including significant weight loss, diarrhea, shortened colon lengths, and fecal blood contamination. Notably, compared with the DSS group, the mice treated with Lut demonstrated a substantial alleviation of weight loss (Figure 1B). Additionally, the DAI of the Lut-treated group significantly reduced throughout the DSS modeling period (Figure 1C). DSS administration caused colon shortening in the mice, an effect that was mitigated following the oral administration of Lut (Figure 1D,E). The spleen is a crucial immune organ that plays a pivotal role in the body’s inflammatory response, and its size is closely correlated with inflammatory activity [37]. The splenic index was significantly higher in the DSS group than in the control group. However, Lut treatment significantly suppressed this increase (Figure 1F). Furthermore, the H&E staining of the colon tissues revealed that Lut significantly reversed DSS-induced colonic inflammatory cell infiltration and tissue damage (Figure 1G), leading to a significant reduction in pathological scores (Figure 1H). Collectively, these data indicate that Lut effectively alleviated the symptoms of DSS-induced colitis in the mice.

### 3.2. Anti-Inflammatory Effect of Luteolin

**Figure 2:**
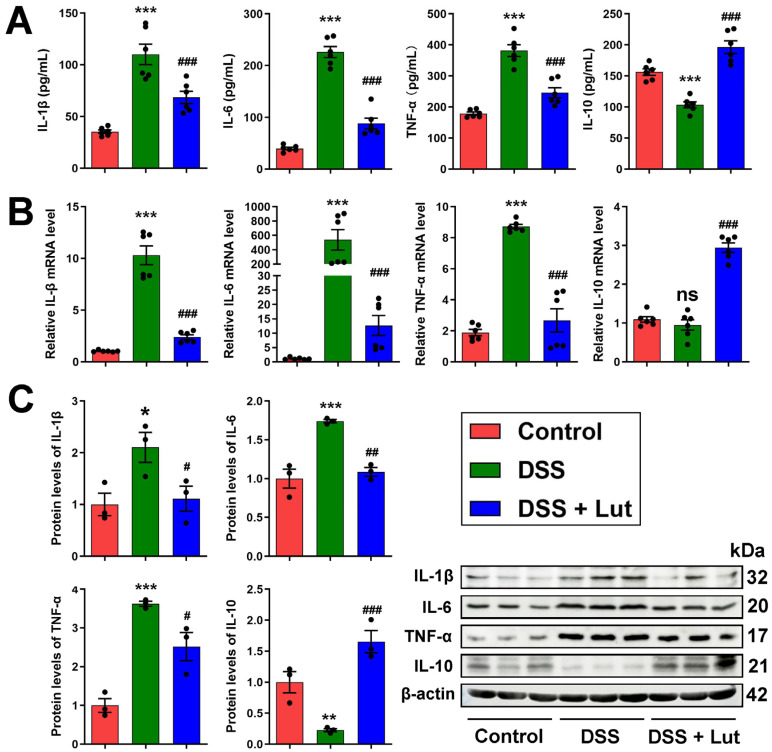
Luteolin treatment regulates the expression of inflammatory cytokines in DSS-induced colitis. (**A**) Lut regulated the release of inflammatory cytokines in the peripheral sera of mice (n = 6). (**B**) Lut modulated the mRNA expression of inflammatory cytokines (*n* = 6). (**C**) Lut altered the protein expression levels of inflammatory cytokines (*n* = 3). * *p* < 0.05, ** *p* < 0.01, *** *p* < 0.001 vs. the control group; # *p* < 0.05, ## *p* < 0.01, ### *p* < 0.001 vs. the DSS group; ns indicates no significant difference vs. the control group.

The abnormal expression of inflammatory mediators plays a crucial role in the pathogenesis of UC [38]. To assess the impact of Lut on the inflammatory response in UC, the release of cytokines in the sera and colon tissues of the mice was detected. Lut significantly inhibited the release of the serum pro-inflammatory cytokines TNF-α, IL-6, and IL-1β and significantly increased the release of the anti-inflammatory cytokine IL-10 in the DSS group mice (Figure 2A). At both the gene and protein levels, the levels of pro-inflammatory cytokines in the colon tissues of the DSS-treated mice were significantly upregulated. However, Lut significantly reduced the expression of these pro-inflammatory cytokines (Figure 2B,C). Notably, although the mRNA levels of IL-10 were not significantly different between the DSS and control groups, the Lut group showed a significant increase in both the IL-10 mRNA and protein levels. In summary, Lut significantly alleviated the inflammatory response in the mice in the DSS group, suggesting its potential as a therapeutic agent for UC by modulating the cytokine balance.

### 3.3. Luteolin Influences the Recovery of the Colon Barrier

**Figure 3:**
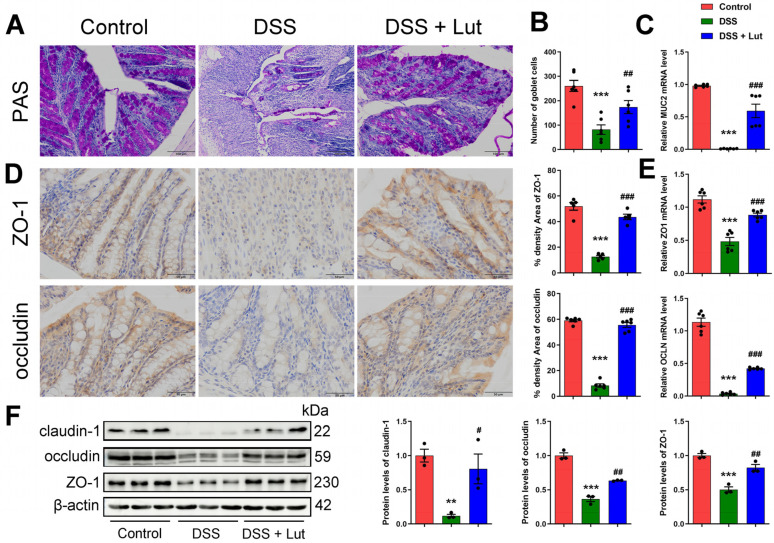
Luteolin attenuates the DSS-induced disruption of colonic barrier integrity in mice. (**A**) Histological analysis of colon sections stained with PAS. Scale bar = 100 μm. (**B**) Statistical analysis of goblet cells based on PAS staining. (**C**) Results of qPCR for the mucin MUC2. (**D**) Immunohistochemical staining was performed to visualize ZO-1 and Occludin proteins. The ratio of positively expressed optical densities was analyzed using Image-J software (version 1.4.3) to quantify the staining intensity. Scale bar = 50 μm. (**E**) The qPCR results of ZO-1 and Occludin. (**F**) The expression of Claudin-1, ZO-1, and Occludin was detected by Western blot (*n* = 3). ** *p* < 0.01, *** *p* < 0.001 vs. the control group; # *p* < 0.05, ## *p* < 0.01, ### *p* < 0.001 vs. the DSS group.

The integrity of intestinal barrier function is a key component in alleviating the symptoms of UC. During this process, TJ proteins play a central role in constructing the epithelial barrier and maintaining epithelial homeostasis [39], whereas intestinal cuprocytes are primarily responsible for synthesizing and secreting mucus, which is essential for maintaining homeostasis in the intestinal environment. Mucin 2 (MUC2) is the most important mucin in the intestinal epithelium and is indispensable for maintaining intestinal homeostasis [40]. Treatment with Lut significantly improved the goblet cell reduction in the DSS group, as detected by PAS staining (Figure 3A,B). Furthermore, MUC2 gene expression detected using qPCR showed that the MUC2 expression in the Lut intervention group was significantly higher than that in the DSS group (Figure 3C). Immunohistochemical analyses demonstrated that the expression of the TJ proteins ZO-1 and Occludin was significantly decreased in the DSS group, whereas their protein expression levels were significantly increased following Lut intervention (Figure 3D). The mRNA levels of ZO-1 and Occludin were detected using qPCR, the results of which showed a consistent trend with the changes in protein levels (Figure 3E). Additionally, the protein expression levels of ZO-1, Occludin, and Claudin-1 were detected using Western blot, and the results were consistent with those of the immunohistochemical analyses (Figure 3F). In conclusion, Lut effectively reversed DSS-induced intestinal barrier damage in the mice and has significant application prospects for the treatment of UC.

### 3.4. Regulatory Effects of Luteolin on Gut Microbiota

**Figure 4:**
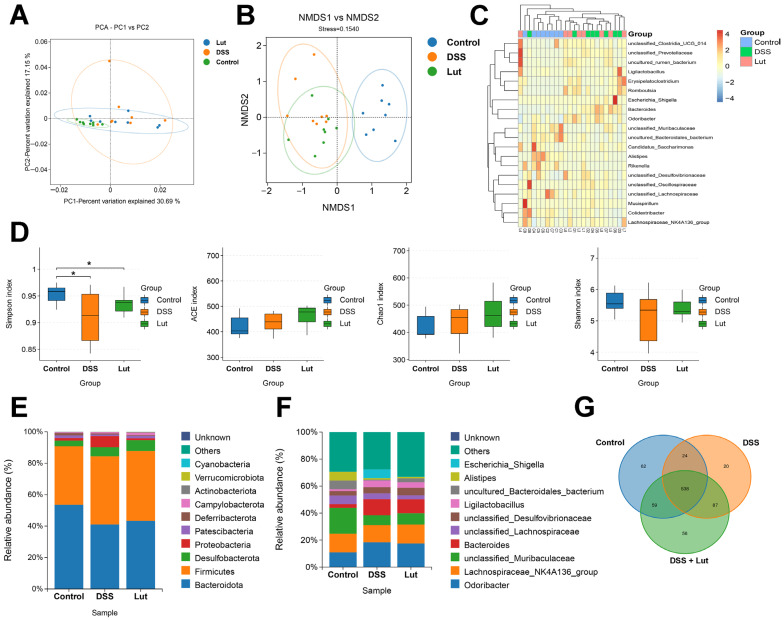
16S rRNA sequencing reveals altered microbiota composition after Luteolin treatment. (**A**) PCA plot of the microbiota. The oval confidence interval was set at 95%. (**B**) NMDS score plot. The oval confidence interval was set at 95%. (**C**) Graphical representation in the form of a heat map illustrating the changes in microbial community categorized at the genus level. (**D**) α-diversity based on the total OTUs. Simpson, ACE, Chao1, and Shannon indices, respectively. * *p* < 0.05 vs. the control group. (**E**) Column charts conveying the gut microbiota at the phylum level. (**F**) Column charts conveying the gut microbiota categorized at the genus level. (**G**) Venn diagram (*n* = 8).

To investigate how Lut affects intestinal microbiota and promotes the establishment of intestinal homeostasis, 16S rRNA amplicon sequencing was used for an in-depth analysis of the microbiota in mouse intestinal feces. The results revealed significant differences in the microbiota between the DSS and control groups, with the Lut group exhibiting an intermediate profile (Figure 4A,B). This suggests that Lut intervention guided the DSS-perturbed gut microbial ecology towards the state observed in the control group. A PCA analysis indicated that the first principal component (PC1) contributed 30.69% of the total variation, and PC2 contributed 17.15% (Figure 4A). The stress value of 0.1540 in the non-metric multidimensional scaling (NMDS) analysis confirmed the reliability of the results (Figure 4B). Additionally, genus-level bidirectional clustering heat maps of species abundance demonstrated better clustering (Figure 4C). Dilution curve analyses revealed that the curves for the three groups tended to flatten with increasing sequencing depth, indicating sufficient sample sequencing (Figure S1A). In terms of diversity, the Simpson index revealed that the microbial diversity of both the DSS and Lut groups was significantly lower than that of the control group. While the diversity in the Lut group was restored, it did not differ significantly from that in the DSS group. In the other index analyses, there were no significant differences between the groups (Figure 4D). However, in the Shannon index analysis, the DSS group exhibited significantly lower microbiota diversity and richness than the control group, with the Lut group being between the two groups (Figure S1B). These findings suggest that Lut intervention restores the diversity and richness of the gut microbiota to a certain extent. Furthermore, the distribution of gut microbes at the phylum and genus levels was analyzed. At the phylum level, *Bacteroidota*, *Firmicutes*, and *Desulfobacterota* were the dominant phyla (Figure 4E). At the genus level, *Odoribacter*, *Lachnospiraceae_NK4A136_group*, *Muribaculaceae*, and *Bacteroides* were the dominant genera (Figure 4F). To visualize the differences in microbiota among the groups, Venn diagrams were used to present the number of gut microbial genera in the different groups (Figure 4G). There were 538 shared genera among the three microbiota groups, of which 62, 20, and 56 genera were exclusive to the control, DSS, and Lut groups, respectively.

### 3.5. Analysis of Intergroup Differences in Intestinal Microbial Communities and Their Correlation with Colonic Inflammatory Manifestations

**Figure 5:**
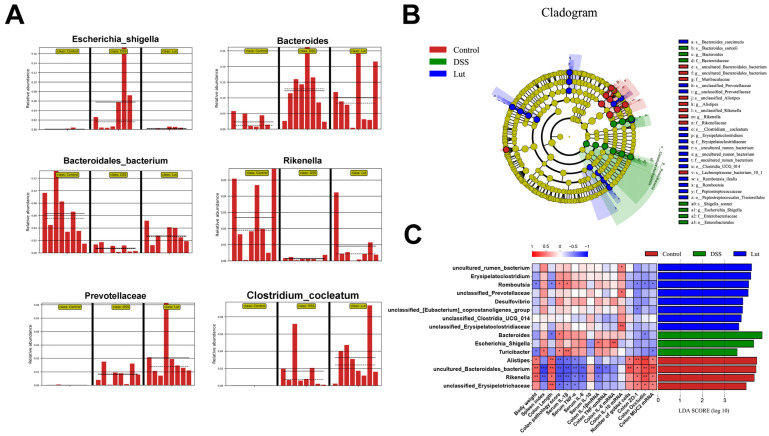
LEfSe analysis of characteristics in the gut microbiota between groups. (**A**) Significantly differential genera and species in the fecal microbiota of different groups after Lut intervention. LEfSe shows its relative abundance distribution in different groups, and the mean and median abundance of the OTUs in each group are identified by solid and dashed lines, respectively. (**B**) Evolutionary branch map. Circles radiating from inside to outside represent taxonomic levels from phylum to species. (**C**) A heat map representing Spearman’s rank correlation coefficients between the gut microbiota and colitis-associated phenotype characteristics for each group (on the left). Analysis of the differential abundance of microbial taxa as identified by LEfSe (on the right). LDA ≥ 4 was used as a threshold value of the characteristic taxon (*n* = 8). * *p* < 0.05, ** *p* < 0.01, *** *p* < 0.001 represent the *p*-value of Pearson’s correlation.

A histogram of the intergroup comparisons of the top 20 bacteria with the smallest *p* values at the gate and genus levels was developed using ANOVA (Figure S2). At the phylum level, the abundance of *Proteobacteria* significantly increased in the DSS group, which was reduced by Lut. At the genus level, the most significant change in bacterial abundance was observed in *unclassified_Muribaculaceae*. LEfSe was used to further dissect the differences in the gut microbiota between the groups. The *Escherichia_shigella* and *Bacteroides* genera significantly increased in the DSS group, whereas Lut supplementation reduced their relative abundance (Figure 5A). In addition, the abundance of *Bacteroidales_bacterium* and *Rikenella* was prominent in the control group, and these taxa exhibited a significant reduction following DSS treatment, which was partially mitigated by subsequent Lut administration. Additionally, as shown in Figure 5B, *Lachnospiraceae_bacterium_10_1*, *Alistipes*, and *Muribaculaceae* were abundant in the control group, whereas Bacteroides were significantly enriched in the DSS group. In the Lut group, *Bacteroides_caecimuris*, *Erysipelatoclostridium*, *uncultured_rumen_bacterium*, and *Romboutsia* were significantly enriched.

To show the correlation between the detected indicators and gut microbiota more intuitively, the heat map was combined with an LDA score bar chart (Figure 5C). *Bacteroidales_bacterium* and *Erysipelotrichaceae*, which were enriched in the control group, exhibited significant positive correlations with colitis indicators, such as colon length, goblet cell number, and TJ protein expression. Conversely, *Bacteroides*, which was enriched in the DSS group, was positively correlated with inflammatory factors and other indicators. However, the bacteria enriched in the Lut group showed weaker correlations with these indices.

### 3.6. Lut Changes the Plasma Metabolic Profiles of Mice

**Figure 6:**
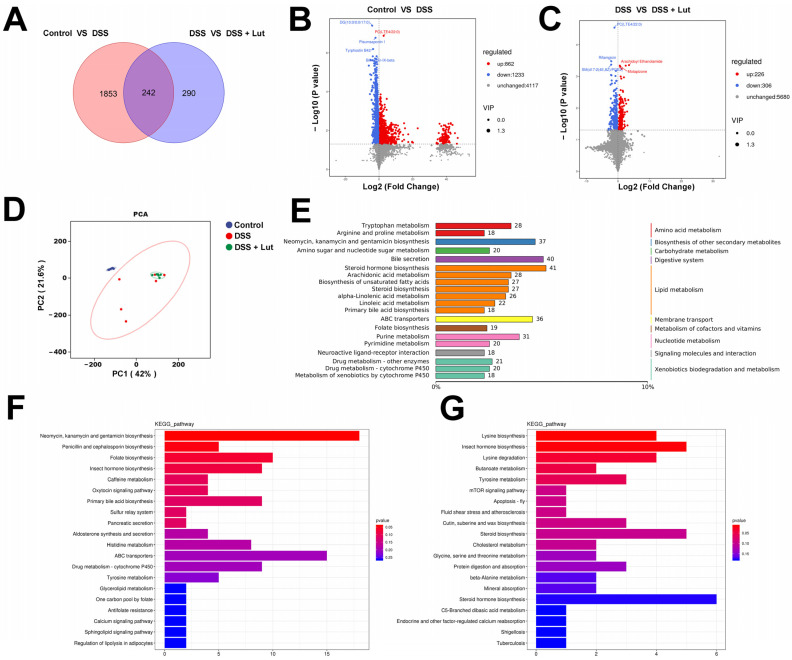
Impacts of Luteolin on plasma metabolic profiles in DSS-induced colitis. (**A**) A Venn diagram illustrating the shared metabolites between the two comparisons: control vs. DSS and DSS vs. Lut. Volcano plots depicting the differential metabolite profiles for (**B**) the control vs. DSS and (**C**) DSS vs. Lut. (**D**) A principal component analysis (PCA) score plot representing all the samples. (**E**) Classification of metabolites based on the KEGG compound database. KEGG pathway enrichment analysis for (**F**) the control vs. DSS and (**G**) DSS vs. Lut (*n* = 6).

Using metabolomics technology, the regulatory effect of Lut on metabolites in mouse plasma was studied in depth, with the aim of identifying candidate target metabolites that may alleviate UC. A total of 2385 known metabolites were identified in plasma samples from the three groups of mice using UPLC-QTOF-MS/MS technology, of which 242 metabolites were in common among the three groups (Figure 6A). Between the control and DSS groups, 2095 differential metabolites were screened, comprising 862 upregulated and 1233 downregulated metabolites. Notably, phosphatidylcholine (PC) (LTE4/22:0) was significantly upregulated in the DSS group, whereas diacylglycerol (DG) (10:0/0:0/17:0), pisum saponin, and tyrphostin B42 were significantly downregulated (Figure 6B). These alterations reflect the metabolic disruptions characteristic of DSS-induced UC pathology. When comparing the DSS and Lut groups, 532 differential metabolites were identified, of which 226 were upregulated and 306 were downregulated. PC (LTE4/22:0) was significantly downregulated in the Lut group, whereas Arachidoyl Ethanolamide and Motapizone were significantly upregulated (Figure 6C). These changes indicate that Lut may ameliorate UC symptoms by modulating the levels of specific metabolites. In addition, inter-sample correlation analyses revealed good biological replicates within the groups (Figure S3A), which was further supported by a PCA, demonstrating significant intergroup differences and confirming the substantial impact of Lut on the plasma metabolic profiles of the mice (Figure 6D). Additionally, OPLS-DA results indicated a high Q2Y of 0.796 for the control/DSS group and 0.3 for the DSS/Lut group, suggesting a closer distance between the DSS and Lut groups compared to the control and DSS groups (Figure S3B). The permutation test of OPLS-DA further validated the reliability of the model, which supported the experimental conclusion (Figure S3C).

**Figure 7:**
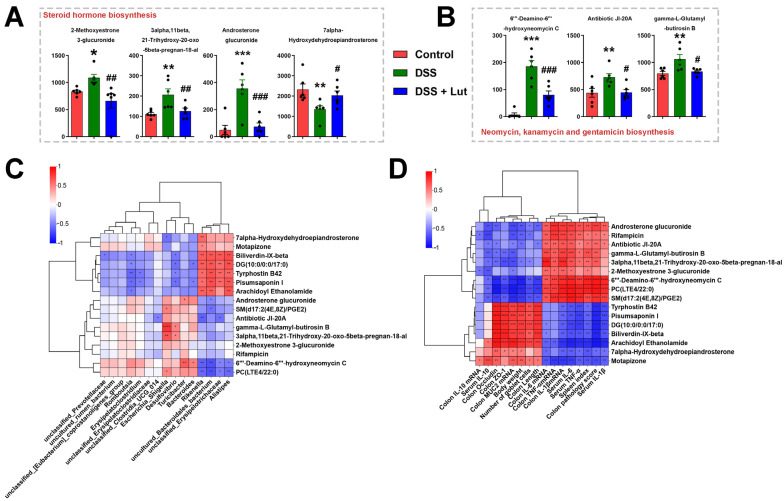
Quantification of important metabolites and their correlation analysis. (**A**) Quantification of metabolites associated with the *steroid hormone biosynthesis* pathway. (**B**) Quantification of metabolites associated with the *neomycin*, *kanamycin and gentamicin biosynthesis* pathway. (**C**) Heat map of Spearman’s correlation analysis of key metabolites and 15 key genera. (**D**) Heat map of Spearman’s correlation analysis of key metabolites and clinical indicators of colitis. * *p* < 0.05, ** *p* < 0.01, *** *p* < 0.001 vs. the control group; # *p* < 0.05, ## *p* < 0.01, ### *p* < 0.001 vs. the DSS group. * *p* < 0.05, ** *p* < 0.01, *** *p* < 0.001, **** *p* < 0.0001 represent the *p*-value of Pearson’s correlation.

Pathway analyses using the KEGG database to annotate all the identified metabolites revealed that the *steroid hormone biosynthesis* pathway was the most enriched with differential metabolites (41 metabolites), followed by the bile secretion pathway (40 metabolites) (Figure 6E). Differential analyses further revealed that DSS treatment affects the metabolic pathway of *neomycin, kanamycin and gentamicin biosynthesis* in mice, with DSS significantly upregulating metabolites within this pathway (Figure 6F). Conversely, Lut treatment downregulated metabolites in the *steroid hormone biosynthesis* pathway and upregulated those in the *lysine biosynthesis* pathway (Figure 6G). To visualize these changes, the metabolite contents in the *steroid hormone biosynthesis* and *neomycin kanamycin and gentamicin biosynthesis* pathways are depicted in Figure 7A,B. Lut significantly downregulated metabolites such as 2-Methoxyestrone-3-glucuronide, 3alpha-11beta-21-Trihydroxy-20-oxo-5beta-pregnan-18-al, and 6′-Deamino-6′-hydroxyneomycin C while significantly increasing 7-alpha-Hydroxydehydroepiandrosterone (Figure 7A,B). In addition, Spearman’s correlation analysis was performed between key metabolites and 15 bacterial genera with LDA ≥ 4 (Figure 7C). The four genera that were significantly enriched in the control group were positively correlated with DG (10:0/0:0/17:0), Tyrphostin B42, and Pisumsaponin. However, they were negatively correlated with PC (LTE4/22:0) and 6′-Deamino-6′-hydroxyneomycin C. In addition, gamma-L-Glutamyl-butirosin B was positively correlated with *Escherichia_shigella*. Spearman’s correlation analysis between key metabolites and the colitis clinical index revealed that Arachidoyl Ethanolamide and Biliverdin-IX-beta were positively correlated with a favorable clinical index and negatively correlated with a poor clinical index (Figure 7D). This suggests that these metabolites play an important role in reducing intestinal inflammation in mice with colitis. In contrast, SM (d17:2 (4E, 8Z)/PGE2) and PC (LTE4/22:0) levels were negatively and positively correlated with favorable and unfavorable clinical indices, respectively. This further highlights the potential roles of these metabolites in the pathogenesis of colitis.

## 4. Discussion

Ulcerative colitis (UC) is a complex autoimmune disease whose precise pathogenesis has not yet been fully elucidated [7]. Currently, it is postulated that disruptions in the intestinal microbiota, driven by environmental factors, serve as the initial trigger for the pathogenesis of UC [8]. However, conventional therapeutic drugs for UC frequently exert additional effects on the diversity and metabolic activity of intestinal flora, thereby limiting their therapeutic effectiveness [41]. Consequently, exploring safe and effective alternative therapies, such as dietary supplementation with naturally low-toxicity botanicals, has emerged as a new strategy for alleviating UC symptoms and modulating gut microbiota imbalances. Lut, a natural flavonoid, reduces inflammation, promotes the repair of damaged tissues, and regulates intestinal microbial balance [30,31,42]. However, the intricate interactions between Lut and various biological processes, including microbial dynamics, metabolism, epithelial barrier function, and inflammatory responses, have not yet been elucidated. In this study, we used a DSS-induced UC mouse model and administered Lut to determine its correlation with UC. Our findings indicated that Lut has the potential to alleviate symptoms, decrease systemic inflammation, and restore the intestinal barrier in mice with colitis. These beneficial effects may be related to alterations in the composition of the intestinal microbiota and regulation of differential metabolites. In summary, this study underscores the potential therapeutic value of Lut in the treatment of UC and offers crucial insights for the further exploration of its mechanisms of action and the development of novel UC treatment strategies.

Numerous studies have confirmed that Lut possesses beneficial anti-inflammatory properties in the context of inflammatory diseases. In a rat model of sodium iodoacetate-induced osteoarthritis (OA), Lut intervention effectively halted cartilage destruction and augmented type II collagen expression [43]. Similarly, in an animal model of acute kidney injury, cerium ion-Lut nanocomplexes exhibited a remarkable capacity to repair damaged renal tissues and mitigate oxidative stress and inflammatory responses [44]. In the present study, Lut (25 mg/kg/day) alleviated clinical symptoms such as weight loss, colon shortening, and pathological damage in UC-inflicted mice and significantly inhibited the expression of pro-inflammatory cytokines in both sera and colon tissues. Furthermore, Lut upregulated the expression of the anti-inflammatory factor IL-10, thereby effectively restoring the colonic barrier damage caused by DSS. Notably, the safe dose of Lut was lower than the effective doses of *dioscin* and *forsythopolyphenol*, which are commonly used to alleviate UC [45,46]. These results suggest that Lut has an effective palliative effect on UC and provide a strong basis for the further exploration of the potential mechanisms by which Lut alleviates UC.

Nutritional interventions can reshape the composition and structure of the intestinal flora, thereby modulating the host immune system, which is a crucial factor in UC pathogenesis [47]. To explore this, we employed 16S microbial diversity sequencing to determine the gut microbiota composition in the mice. Previous studies have documented a reduction in gut microbiota diversity in mice with DSS-induced UC [48]. However, our fecal microbiome analysis revealed no significant changes in fecal microbial α diversity among the three mouse groups. Similar results have been reported previously. This suggests that the changes in colitis symptoms were not attributed to drastic shifts in the gut microbiota diversity. Consequently, we delved deeper into the microbiota composition at a more granular taxonomic level. At the phylum level, our analysis revealed a significant increase in the abundance of *Proteobacteria* in the DSS group, which was decreased in the Lut group. This aligns with the impact of Scutellaria baicalensis Georgi polysaccharides on the intestinal flora of UC model mice [15]. Notably, *Proteobacteria* include many potential pathogens [49]. Further analyses showed that the abundance of *Escherichia_Shigella* in *Proteobacteria* was significantly increased in the DSS group, which was effectively inhibited after Lut intervention. This was similar to the effect of deferasirox on intestinal *Escherichia_Shigella* in the UC-inflicted mice. *Escherichia_Shigella* has been linked to alcoholic cirrhosis, tuberculous meningitis, and other inflammatory diseases, as well as various intestinal diseases [14,50]. In addition, *Bacteroides* were significantly upregulated in the DSS group. *Bacteroides* usually maintain a complex and relatively beneficial relationship with the host in the gut, but the roles of different *Bacteroides* species in UC may differ. For example, the capsular polysaccharides of *Bacteroides fragilis* are thought to protect against UC [51], whereas other species may exacerbate inflammation by stimulating IL-17 production in the gut [52]. This could explain why the *Bacteroides* abundance was not significantly downregulated under Lut intervention. Moreover, potentially beneficial bacteria enriched in the control group, such as *Bacteroidales_bacterium* and *Rikenella* under Muribaculaceae, were significantly reduced after DSS treatment, whereas Lut treatment partially restored their abundance. Notably, *Rikenella* aids in the formation of intestinal epithelial cells and is considered the most effective antidiarrheal probiotic. *Lactiplantibacillus plantarum* BW2013 similarly upregulated the abundance of intestinal *Rikenella* in a mouse model of UC [53]. Notably, *Prevotellaceae* and *Clostridium_cocleatum*, which were almost undetectable in the control group, significantly increased after Lut intervention. *Prevotellaceae* are well-known for their beneficial properties [54,55]. However, no direct correlation has been established between *Clostridium_cocleatum* and UC. In the treatment of colorectal cancer, the use of probiotic powder led to a decrease in the abundance of *Clostridium_cocleatum* [56]. Furthermore, *Alistipe*, *Bacteroidales_bacterium*, *Erysipelotrichaceae*, and *Rikenella* were positively correlated with a positive symptom index and negatively correlated with a negative symptom index, suggesting their potential benefits. However, it has also been reported that *Alistipe* is significantly enriched in colorectal cancer mouse models with potential pathogenic characteristics [57]. This may be related to complex interactions between microorganisms in different spaces and environments. No significant difference was observed in the relative abundances of *Lactobacillus* and *Helicobacter*. Although Lactobacillus is generally considered a beneficial genus, it also inhibits inflammatory responses under various inflammatory conditions [58]. *Helicobacter* infection is often considered harmful and may play an important role in the occurrence of UC [59]. In addition, *Escherichia_Shigella* was positively correlated with inflammatory factors IL-1β and IL-6, further validating its detrimental role in UC pathogenesis. In conclusion, Lut treatment increased the levels of beneficial bacteria and decreased those of pathogenic bacteria in the gut microbiota, thereby promoting anti-inflammatory cytokine release and inhibiting pro-inflammatory cytokine production. This mechanism may be pivotal for the anti-inflammatory effects of Lut in mouse colitis models.

The gut microbiota exert considerable influence on host metabolism. There was a significant positive correlation between *Escherichia_Shigella* and phosphatidylcholine (PC), particularly in relation to LTE4/22:0. The critical role of PC in the interactions between pathogenic microorganisms and their hosts has been extensively studied. For example, human pathogens, such as *Brucella abortus* and *Legionella pneumophila*, rely on PC to attain full virulence [60]. Consequently, we hypothesized that PC plays an important role in the pathogenic effects of *Escherichia_Shigella* in mice. However, it is worth noting that PC has also been viewed as a potential therapeutic agent for IBD because of its protective effect on the mucosa [61]. Furthermore, gamma-L-Glutamyl-butirosin B exhibited a positive correlation with *Escherichia_Shigella*. Although Butirosin, an aminoglycoside antibiotic produced by *Bacillus circosus*, was considered, our study did not reveal any differences in the gut microbiota *Bacillus circosus* [62]. Additionally, antibiotic-related metabolites, such as 6′-Deamino-6′-hydroxyneomycin C and Antibiotic JI-20A, were enriched in KEGG signaling pathways for *neomycin, kanamycin and gentamicin biosynthesis* [63]. These metabolites were also significantly upregulated in the DSS-treated mice. These findings suggest that the mouse gut microbiota significantly affects the body’s metabolite levels through changes in itself and its metabolites. Moreover, the *steroid hormone biosynthesis* pathway was significantly downregulated in the Lut-treated mice, indicating that DSS and Lut influence reproduction-related hormone metabolism in mice. Notably, motapizone, a Phosphodiesterase-3 (PDE3) inhibitor, has demonstrated the potential for treating severe pulmonary hypertension and inhibiting lipopolysaccharide (LPS)-induced cytokine release from alveolar macrophages [64,65]. Arachidoyl-ethanolamide (AEA), an endocannabinoid, is associated with various physiological diseases, including obesity, liver diseases, nervous system diseases, and inflammation [66,67]. In the present study, Motapizone and AEA levels decreased with DSS treatment, whereas Lut treatment restored these levels. This suggests that Lut alleviates UC in mice by increasing the metabolism of Motapizone and AEA. As an inflammatory mediator, the expression of SM (d17:2 (4E, 8 Z)/PGE2) increases during various inflammatory reactions [68]. In our study, Lut reduced the high levels of expression in the DSS group, suggesting that PGE2 may be a key therapeutic target for IBD. Tyrphostin B42 (AG490), a Janus Kinase (JAK)-2 protein tyrosine kinase inhibitor, has a broad range of anti-inflammatory activities [69,70]. However, in our study, DSS treatment significantly reduced Tyrphostin B42 levels, whereas Lut treatment did not.

Correlation analyses between clinical indicators and differential metabolites not only deepen our understanding of metabolites that play a key role in the occurrence and development of UC in mouse models but also provide new insights into the pathogenesis of UC and potential targets for the development of new therapeutic strategies. However, there are some limitations to this study as well. For instance, further evidence should be provided to establish the correlation between the relevant microbial taxa and metabolic products and UC. Additionally, it is necessary to investigate whether these factors play a pivotal role as targets in the process of Lut alleviating UC. In addition, this study did not assess the liver parameters in mice with enteritis. Whether DSS can induce inflammation in the liver tissue through the enterohepatic circulation, and whether Lut can mitigate this potential effect, are research directions that should be given attention in future studies.

## 5. Conclusions

In this study, we investigated the intrinsic relationship between the gut microbiota and plasma metabolic profiles to elucidate the mechanisms underlying the effectiveness of Lut in alleviating DSS-induced UC in a mouse model. Our findings revealed that Lut administered at a dose of 25 mg/kg significantly ameliorated colitis symptoms in mice. It effectively inhibited the colonic and systemic inflammatory responses and promoted the repair of colonic barrier damage. Notably, the effect of Lut on gut microbiota diversity and richness was negligible. Instead, its anti-inflammatory effect was mainly mediated by inhibiting the growth of pathogenic bacteria, such as *Escherichia_Shigella*, and increasing the abundance of beneficial bacteria, including *Muribaculaceae*, *Rikenella*, and *Prevotellaceae*. Further analyses revealed several metabolites, such as Tyrphostin B42 and AEA, which exhibited a positive correlation with beneficial bacteria and may possess anti-inflammatory or protective effects. Consequently, we identified metabolites such as PC that were positively associated with pathogenic bacteria such as *Escherichia_Shigella*, suggesting potential biological targets for future UC treatment strategies. This study not only deepens our understanding of the mechanisms of action of Lut in the treatment of UC but also provides a solid theoretical and experimental basis for the development of therapeutic strategies using Lut to intervene in the occurrence and development of UC.

## Figures and Tables

**Table 1:** Detailed scoring method of the disease activity index.

Score	Weight Loss	Stool Characteristics	Degree of Hematochezia
0	<1%	Normal	Negative
1	1–5%	Formed but adherent	Weak positive
2	5–10%	Semi-formed/soft	Positive
3	10–20%	Liquid-like consistency but does not adhere to the anus	Strong positive
4	>20%	Adheres to the anus	Visible blood in stool

## Data Availability

The data that support the findings of this study are available from the corresponding author upon reasonable request.

## Supplementary Materials

The following supporting information can be downloaded at https://www.mdpi.com/article/10.3390/nu17020203/s1: Table S1: Detailed scoring method of histology; Table S2: Detailed information on primers used for qPCR of 8 genes; Figure S1: Supplementary Materials for microbiological analysis; Figure S2: Supplementary Materials for analysis of differences between the microbiome; Figure S3: Supplementary Materials for plasma metabolomics analysis.
