# Supplementary material for: Luteolin Alleviates Ulcerative Colitis in Mice by Modulating Gut Microbiota and Plasma Metabolism
Source: Nutrients. 2025 Jan 7;17(2):203. doi: 10.3390/nu17020203 (PMC11768085; doi:10.3390/nu17020203)
Supplement: Supplementary file 1 [file nutrients-17-00203-s001.zip › nutrients-3402423-supplementary.pdf]

Supplementary Materials

Table S1. Detailed scoring method of histology

Score	The severity of inflammation	Degree of tissue damage
0	Absence of inflammation	Normal
1	Inflammatory infiltrates were occasionally seen	A small number of goblet cells were lost<br>Discrete lymphoepithelial lesions
2	The mucosal layer was moderately inflammatory	There was a large loss of goblet cells<br>The surface mucosa was erosive
3	There was extensive inflammatory infiltration of the muscularis mucosa	A small number of crypts were missing<br>The mucosa was edematous and thickened
4	There was extensive inflammatory infiltration of the submucosa	There was a large area of crypt loss<br>Extended deeper structural damage

Table S2. Detailed information on primers used for qPCR of 8 genes.

Gene	Primer name	Sequences (5′-3′)	Tm(°C)
GAPDH	GAPDH-F	GATGGTGAAGGTCGGAGTGAAC	60
	GAPDH-R	GTCATTGATGGCGACGATGT	
IL-1B	IL-1B -F	GGATGAGGACATGAGCACCT	60
	IL-1B -R	GGAGCCTGTAGTGCAGTTGT	
IL-6	IL-6-F	TACCACTTCACAAGTCGGAGGC	60
	IL-6-R	CTGCAAGTGCATCATCGTTGTTC	
IL-10	IL-10-F	CGGGAAGACAATAACTGCACCC	60
	IL-10-R	CGGTTAGCAGTATGTTGTCCAGC	
TNF-α	TNF-α-F	GGTGCCTATGTCTCAGCCTCTT	60
	TNF-α-R	GCCATAGAACTGATGAGAGGGAG	
MUC2	MUC2-F	GCCCACCTCACAAGCAGTAT	60
	MUC2-R	GTCATAGCCAGGGGCAAACT	
ZO-1	ZO-1-F	GTTGGTACGGTGCCCTGAAAGA	60
	ZO-1-R	GCTGACAGGTAGGACAGACGAT	
Occludin	Occludin -F	CAGGTGAATGGGTCACCGAG	60
	Occludin -R	CCAAGATAAGCGAACCTGCC	

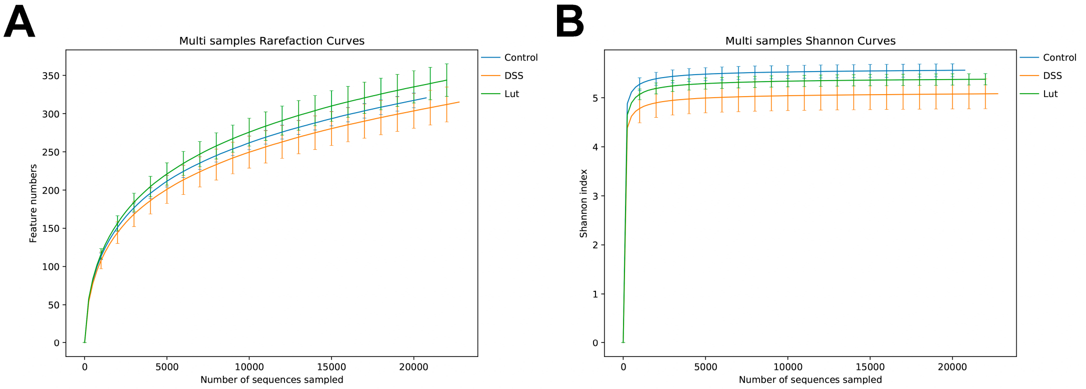

Figure S1. Supplementary materials for microbiological analysis. (A) Rarefaction curve of the microbiome. (B) Shannon index curve of the microbiome.

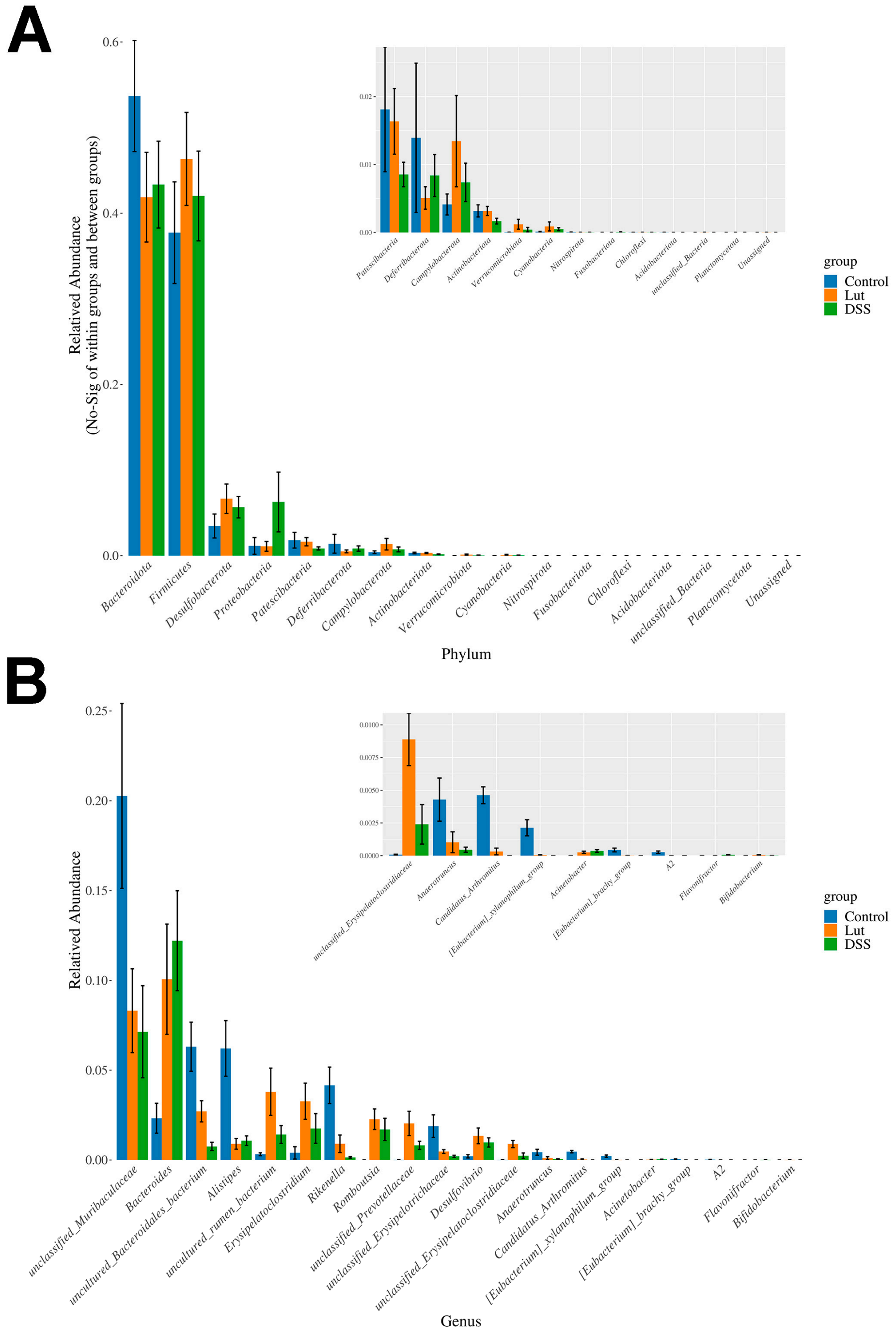

Figure S2. Supplementary materials for analysis of differences between the microbiome. A histogram of the intergroup comparisons of the top 20 bacteria with the smallest *P* values at the (A) phylum and (B) genus levels was developed using ANOVA.

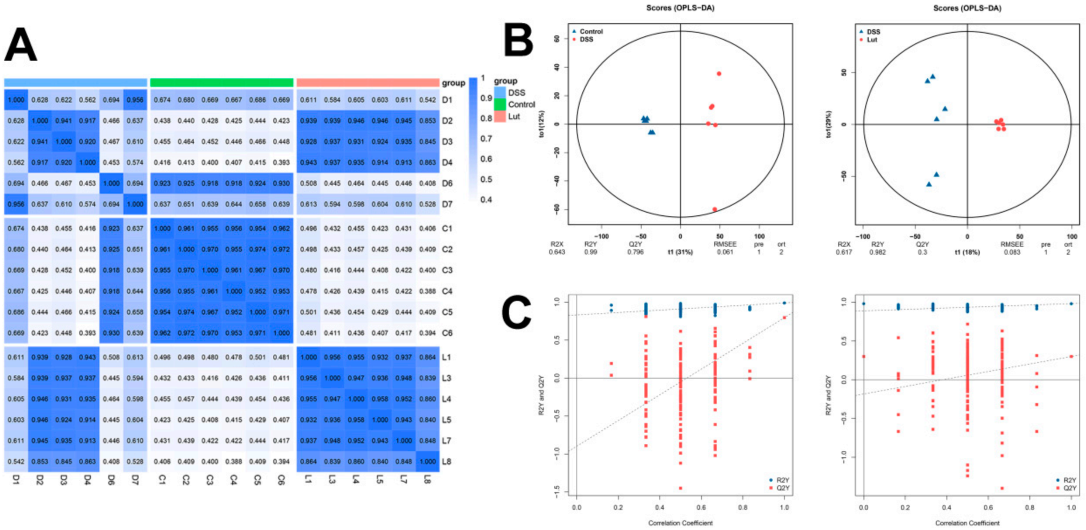

Figure S3. Supplementary materials for plasma metabolomics analysis. (A) Sample correlation analysis of metabolomics. (B) Orthogonal Partial Least Squares Discriminant Analysis. (C) Permutation test of Orthogonal Partial Least Squares Discriminant Analysis.
